# Use of NQO1 status as a selective biomarker for oesophageal squamous cell carcinomas with greater sensitivity to 17-AAG

**DOI:** 10.1186/1471-2407-14-334

**Published:** 2014-05-15

**Authors:** Katie E Hadley, Denver T Hendricks

**Affiliations:** 1 Division of Medical Biochemistry, Department of Clinical Laboratory Sciences, Faculty of Health Sciences, University of Cape Town, Anzio Road, Observatory, Cape Town 7925, South Africa

## Abstract

**Background:**

Oesophageal squamous cell carcinoma (OSCC) is a major health burden in Sub-Saharan Africa, and novel chemotherapies are urgently required to combat this disease. The heat shock protein 90 (HSP90) inhibitor 17-N-allylamino-17-demethoxygeldanamycin (17-AAG) has previously been proposed as a possible candidate drug. NADPH quinone oxidoreductase 1 (NQO1) is known to increase the potency of 17-AAG, therefore we investigated the effects of 17-AAG in OSCC cell lines in the context of their NQO1 status.

**Methods:**

We used MTT assays to compare the sensitivity of a panel of OSCC cell lines to 17-AAG. Western blotting, and RT-PCR were used to investigate NQO1 protein and mRNA levels, while an RFLP approach was used to investigate the NQO1 C609T SNP.

**Results:**

Expression of NQO1 markedly increased sensitivity to 17-AAG in the OSCC cell lines, while normal fibroblasts, which expressed HSP90 at much lower levels, were more resistant to 17-AAG. In isolation, neither the C609T SNP, nor NQO1 mRNA levels was an accurate predictor of NQO1 protein levels.

**Conclusions:**

Since NQO1 greatly enhances the anti-cancer effects of 17-AAG, this could be used as a selective marker for patients that would benefit most from 17-AAG chemotherapy at low doses. Testing for the presence of the C609T SNP in both alleles could be used as a screen to exclude potentially poor responders to 17-AAG treatment at low dosages.

## Background

OSCC presents a major health burden in Sub-Saharan Africa, and novel chemotherapies are urgently needed to combat this disease. HSP90 has been shown to be overexpressed in a number of cancers, and presents an attractive target for anti-cancer therapy, as it plays a central role in contributing to the maintenance of a number of the characteristic hallmarks of cancer cells, by chaperoning key proteins, and maintaining active conformations of signalling proteins, reviewed in [1]. These include important signalling proteins like EGFR and IGF1-R that have been implicated in sustaining the neoplastic phenotype in OSCC [2].

Different HSP90 inhibitors have been shown to have promise as chemotherapeutics.

These include the family of benzoquinone ansamycins (BA’s), such as geldanamycin and its derivatives 17-AAG and 17-DMAG. It has been suggested that 17-AAG could be useful for treatment of OSCC [2]. There are several drawback to this class of drugs, which are reviewed in [3], most notably the induction of hepatotoxicity. This results from one electron reduction by members of the cytochrome p450 family. This reaction results in unstable intermediates, damaging the tissue, which manifests as severe hepatotoxicity [4]. On the other hand, the benzoquinone ansamycins can also undergo two electron reduction by the enzyme NADPH quinone oxidoreductase 1 (NQO1), which results in a compound with higher affinity for HSP90, which is therefore a more potent inhibitor [5,6].

The gene encoding NQO1 has been found to contain a single nucleotide polymorphism at position 609. The C609T SNP causes a proline to serine mutation at position 187 [7], allowing ubiquitination of NQO1 and reducing stability of the protein [8]. Thus, the SNP is effectively a null mutation, as patients homozygous for T at position 609 will express NQO1 that is rapidly degraded by the proteasome.

We aimed to examine the potential of 17-AAG as a chemotherapeutic drug for OSCC, using a panel of cell lines with different NQO1 levels. Results indicated that NQO1 status could be an important determining factor in tumour response to 17-AAG. We next investigated whether the presence of NQO1 enzyme could be predicted with either the absence of the C609T SNP, or expression levels of NQO1 mRNA. Although neither factor alone was sufficient, SNP analysis could allow exclusion of a cohort of NQO1-negative patients who would be less sensitive to 17-AAG.

## Methods

### Cell lines, plasmids, transfections and drugs

17-AAG was purchased from Calbiochem. The human OSCC cell lines WHCO1 and WHCO6, derived from South African patients, were a gift from Prof R. Veale, and described in [9]. The Kyse cell lines were purchased from DSMZ, Germany. All cells were grown in DMEM with 10% FCS, in the presence of penicillin and streptomycin. The plasmids for overexpression of NQO1 (pEFIRES-empty, and pEFIRES-NQO1) were a kind gift from Yosef Shaul (Weizmann Institute of Science) [10]. Cells were transfected using Transfectin (BioRad) and transfected cells were selected using puromycin (Calbiochem). Pools of stably transfected cells were maintained in 1.5 μg/ml puromycin.

### MTT assay

Cells were plated in 96 well plates at a density of 5000 cells per well. The following day, cells were treated with drug at different concentrations. After 2 or more days of incubation, 10 μl of sterile MTT solution (5 mg/ml in PBS) was added to each well, and plates were incubated for 4 hours. Thereafter, 100 μl of solubilisation reagent (10% SDS, 0.01M HCl) was added to each well. Plates were incubated at 37°C overnight, before the absorbance was measured at 595 nm.

### Western blotting

Proteins were harvested in RIPA buffer, and sonicated for 10s. Protein concentration was calculated using the BCA kit (Pierce). Equal amounts of protein were separated on a polyacrylamide gel, and transferred to a nitrocellulose membrane (Amersham). Membranes were blocked in 5% fat free milk powder, before incubation with the following primary antibodies: NQO1 A180 (sc32793); GAPDH 0411 (sc47424); β tubulin H235 (sc9104); PARP 1/2 H250 (sc7150) (all from Santa Cruz Biotechnology).

### SNP analysis

Genomic DNA was harvested from cell lines using Qiazol, according to the user defined protocol provided on the manufacturer’s website. PCR was performed using Amplitaq Gold (Applied Biosystems), and primer sequences from [11]. PCR products were purified using Wizard SV Spin columns (Promega) before being digested overnight with Hinf1 (Thermo Scientific). Digested DNA fragments were analysed by polyacrylamide gel electrophoresis, staining with ethidium bromide.

### Quantitative RT-PCR

Total RNA was harvested from cells at approximately 60- 80% confluency using the Qiazol reagent (Qiagen), according to the manufacturer’s instructions. After agarose gel electrophoresis to confirm RNA integrity, 1μg was reverse transcribed using random hexamer primers, and Impromtu RTase (Invitrogen). cDNA was submitted to quantitative RT-PCR using Sybr-fast reaction mix (Kapa Biosystems), and primers for HSP90α (F: TGAGGACAGACACAGGTGAAC 3′ and R: TGGTCCAGATGGGCTTTGTT 3′) NQO1 (F: TGAAGAAGAAAGGATGGGAGG3′ and R: AGGGGGAACTGGAATATCAC 3′) and β actin (F: AGGAAGGAAGGCTGGAAGAG 3′ and R: ATCGTGCGTGACATTAAGGAG3′). β actin was used as a housekeeping gene. Relative expression was calculated using comparative Ct values. Results of two to three independent experiments were pooled.

### Statistical analysis

GraphPad Prism software was used for statistical analysis, as indicated in figure legends. For MTT dose response assays, absorbance values were analysed by nonlinear regression, with a sigmoidal curve (variable slope), allowing calculation of the IC_50_ value. Dose response experiments were repeated several times in each cell line, and data were pooled to give a more accurate estimation of the IC_50_ and 95% confidence intervals around the value.

## Results

### NQO1 enhances sensitivity of OSCC cell lines to 17-AAG

**Figure 1:**
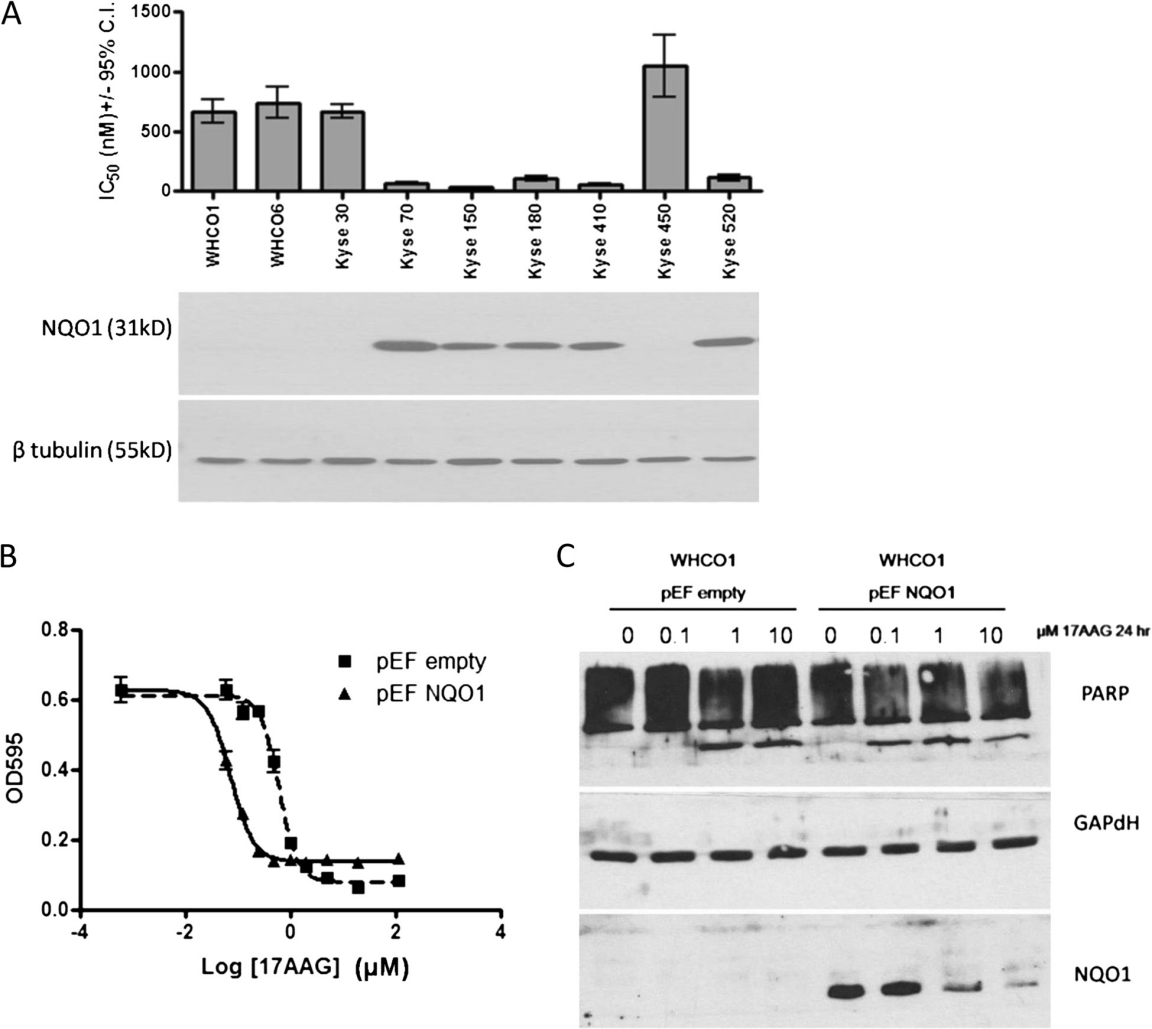
**NQO1 increases sensitivity of OSCC cell lines to 17-AAG. (A)** OSCC cell lines were analysed by dose response MTT assay, and IC_50_ concentrations were established for each cell line, by pooling 2 to 6 independent experiments. The IC_50_ value is presented in the histogram, with error bars representing the 95% confidence interval around this value. The lower panel shows a Western blot of NQO1 levels, with β tubulin as a loading control. The Western blot is representative of at least two independent experiments. **(B)** WHCO1 cells were stably transfected with pEF-empty or pEF-NQO1. Dose response curves were performed in triplicate (error bars represent SEM). Results are representative of two independent experiments. **(C)** Cells were treated with different concentrations of 17-AAG for 24 hr, and protein was harvested and analysed for PARP cleavage by Western blot. NQO1 overexpression was also confirmed by Western blot. Results are representative of two independent experiments.

We analysed the response of a panel of OSCC cell lines to 17-AAG. Using dose response MTT assays, we established the IC_50_ concentrations of 17-AAG for each cell line. We noticed that all the cell lines in the panel were relatively sensitive to 17-AAG, with IC_50_ values in the sub-micromolar range (Figure 1). However, five of the OSCC cell lines were significantly more sensitive, with IC_50_ values below 120 nM. On further investigation, we found that the sensitivity to 17-AAG correlated very well with endogenous expression of NQO1, as detected by Western blotting (Figure 1A, bottom panel). Cell lines with detectable levels of endogenous NQO1 were markedly more sensitive to 17-AAG.

In order to confirm that the levels of NQO1 were indeed responsible for the differences in sensitivity to 17-AAG, we generated stable cell lines overexpressing NQO1 (WHCO1 pEF-NQO1) or the empty vector (WHCO1 pEF-empty). Overexpression of NQO1 was confirmed by Western blotting, and NQO1 levels were found to be similar to the levels of endogenous NQO1 in the cell lines in which NQO1 was detectable (Additional file 1: Figure S1). Analysis of the sensitivity of the stable cell lines to 17-AAG revealed that overexpression of NQO1 resulted in increased sensitivity to the drug, reducing the IC_50_ value of the cells from 0.618 μM (WHCO1 pEF-empty) to 0.0738 μM, which is in line with the IC_50_ values of cell lines that express detectable NQO1 (Figure 1A). We further confirmed the increased sensitivity of the cells by investigating PARP cleavage, a marker of apoptosis, in response to 17-AAG. While WHCO1 cells transfected with empty vector only exhibited PARP cleavage after treatment with 1 μM 17-AAG for 24 hours, NQO1 transfected cells exhibited PARP cleavage at the lower concentration of 0.1 μM 17-AAG (Figure 1C ).

We noted that NQO1 protein levels decreased in the presence of increasing concentrations of 17-AAG. A similar effect was observed with endogenous NQO1 in Kyse 70 and Kyse 150 cells (Additional file 2: Figure S2). However, we did not detect a significant downregulation of NQO1 mRNA brought about by treatment with 17-AAG (Additional file 2: Figure S2), suggesting that the observed downregulation at the protein level is a post-transcriptional event.

**Figure 2:**
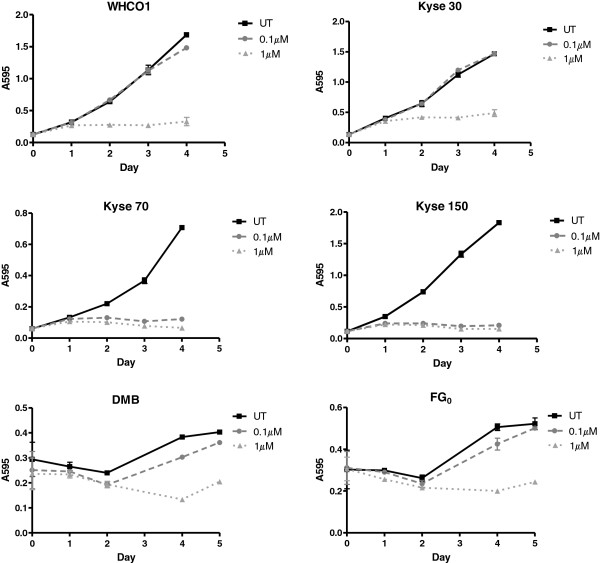
**Sensitivity of OSCC cell lines and normal fibroblasts to 17-AAG.** OSCC cell lines were plated in 96 well plates, and their proliferation was measured over several days in the presence of increasing concentrations of 17-AAG using a MTT assay. Each point was performed in triplicate, and error bars represent standard deviation. Results are representative of two independent experiments.

We selected cell lines with either detectable or undetectable levels of endogenous NQO1, and examined their proliferation over several days in the presence of increasing concentrations of 17-AAG (Figure 2). Although none of the cell lines showed proliferation in the presence of 1 μM 17-AAG, we observed a distinct dichotomy between those OSCC cell lines which expressed NQO1 (Kyse 70 and Kyse 150), which did not proliferate in the presence of 0.1 μM 17-AAG, and those in which NQO1 was not detectable (WHCO1 and Kyse 30), which displayed proliferation levels similar to untreated cells in the presence of 0.1 μM 17-AAG. Western blotting for PARP cleavage as a marker of apoptosis showed that at 0.1 μM 17-AAG, apoptosis was induced within 24 hr of treatment in Kyse 150, and 72 hr of treatment in Kyse 70 (Additional file 3: Figure S3A). No induction of PARP cleavage was detectable in WHCO1 or Kyse 30 at this concentration of 17-AAG over a similar time frame (Additional file 3: Figure S3 B).

**Figure 3:**
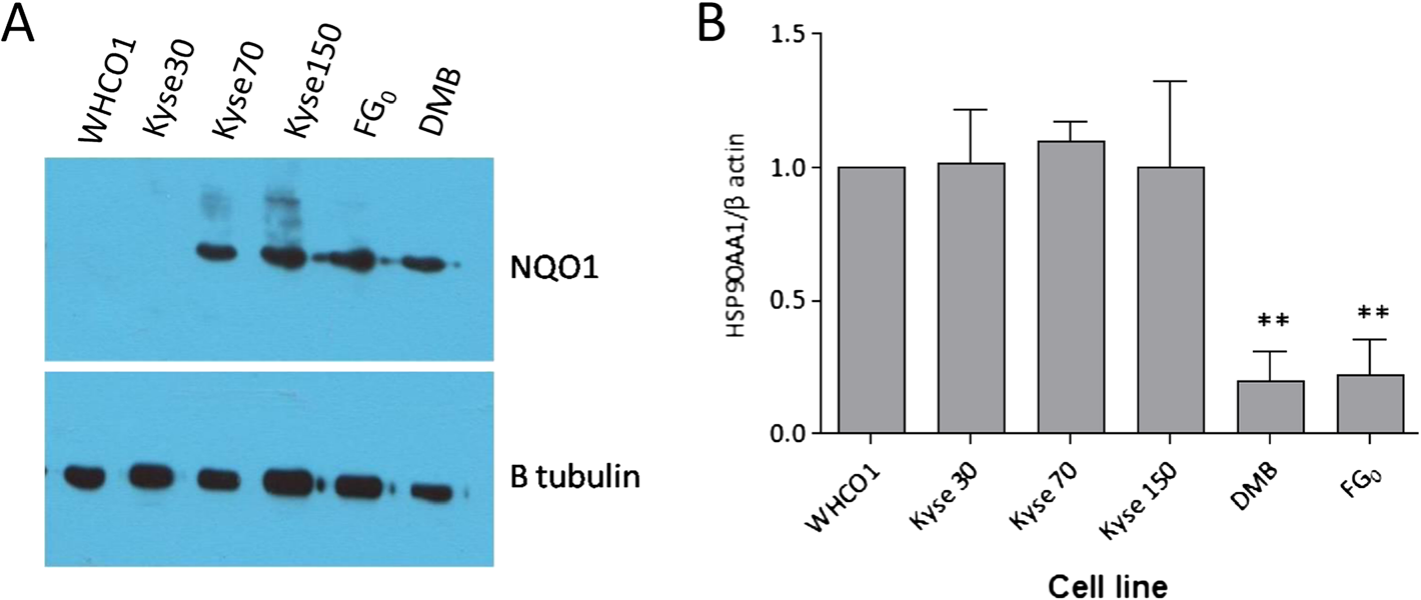
**NQO1 levels and HSP90AA1 expression in OSCC cell lines and normal fibroblasts. (A)** Protein lysates of OSCC cell lines and normal fibroblasts (DMB and FG_0_) were analysed by Western blot for endogenous levels of NQO1. Results are representative of 2 independent experiments. **(B)** Real time PCR was used to measure levels of HSP90AA1 mRNA relative to the housekeeping gene β actin, in a panel of OSCC cell lines and normal fibroblasts. Histogram shows pooled results of 2- 3 independent experiments, analysed by one-way ANOVA, with Dunnet’s post-test relative to WHCO1.

Interestingly, the normal fibroblasts DMB and FG_0_, were relatively unaffected by the presence of 0.1 μM 17-AAG, and proliferated at a similar rate to untreated cells (Figure 2). This is despite their having detectable levels of the 17-AAG metabolising enzyme NQO1, similar to the levels observed in Kyse 70 and Kyse 150 (Figure 3A). This highlights the selectivity of 17-AAG for cancer cells, presumably due to the increased reliance of cancer cells on HSP90 [12]. As expected, we observed that the expression of HSP90 (α subunit) is significantly higher in the OSCC cell lines tested than the normal fibroblasts (Figure 3B), indicative of their increased reliance on HSP90 as a chaperone. This suggests that in NQO1 expressing patients, treatment with a low dose of 17-AAG could still selectively target cancer cells and have minimal effects on normal cells, even though they may express NQO1.

### NQO1 protein levels in OSCC cell lines depend on C609T SNP and expression levels of NQO1 mRNA

**Figure 4:**
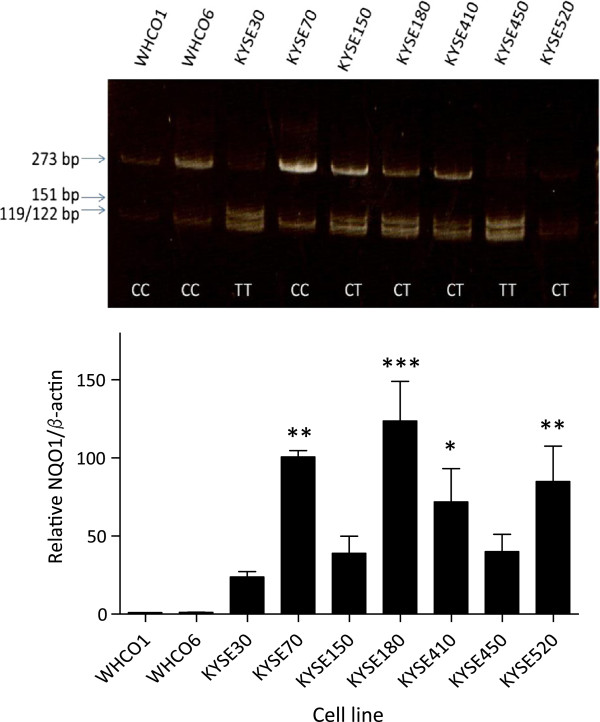
**NQO1 C609T SNP and NQO1 mRNA levels contribute to endogenous levels of NQO1 protein.** Genomic DNA from a panel of OSCC cell lines was amplified using primers spanning the C609T SNP. PCR products were digested with HINf1α, and analysed by polyacrylamide gel electrophoresis to establish the presence of the SNP. (B) Total RNA was extracted from cells at approximately 80% confluency. RNA was reverse transcribed and the abundance of NQO1 mRNA was analysed relative to β actin mRNA using real time PCR. Histogram shows pooled results of 2- 3 independent experiments, analysed by one-way ANOVA, with Dunnet’s post-test relative to WHCO1.

Since the presence of NQO1 was an indicator of high sensitivity to 17-AAG, we postulated that this could be a useful marker of a patient’s suitability for treatment with low doses of 17-AAG. We sought to investigate whether the presence or absence of the NQO1 C609T SNP could allow rapid identification of cell lines with high NQO1 levels, in the hope that this may ultimately be extended to a clinical setting, for selection of patients who would likely respond better to 17-AAG. We used an RFLP approach to genotype the panel of cell lines used (Figure 4). We found that all of the cell lines in which NQO1 was detectable had at least one WT allele (CC or CT). Two cell lines homozygous for the C609T SNP (Kyse 30 and Kyse 450) did not express detectable NQO1, which is consistent with this SNP allowing increased turnover of the nascent protein. Unexpectedly, we observed that two cell lines (WHCO1 and WHCO6) with undetectable NQO1 levels, were homozygous for the wild-type (C) allele. Thus in these cell lines, the absence of detectable NQO1 could not be accounted for by more rapid protein degradation caused by the C609T SNP.

In an attempt to explain this unexpected result, we examined NQO1 mRNA expression in the panel of OSCC cell lines using real time PCR. We found that WHCO1 and WHCO6 expressed lower levels of NQO1 mRNA than the other cell lines with at least one C allele (approximately 50-100 fold lower than Kyse 70, 150, 180 and 520). The expression of NQO1 in WHCO1 and WHCO6 was also approximately 25 times lower than in the two cell lines identified as homozygous for the C609T SNP. The lower levels of NQO1 mRNA could possibly account for the undetectable levels of endogenous NQO1 protein in WHCO1 and WHCO6.

## Discussion

Our results show a clear correlation between NQO1 levels and sensitivity to 17-AAG as expected [5]. Although NQO1 activation is considered necessary for 17-AAG activity [3], even OSCC cell lines without detectable NQO1 showed considerable sensitivity to 17-AAG, with IC_50_ concentrations around 1μM. Although this might lead one to overestimate the promise of 17-AAG as a chemotherapeutic option for OSCC, one must be mindful of the limitations of this in vitro study. Firstly, we have not measured the negative side effects attributed to the drug. Indeed, a recent clinical trial of 17-AAG found that the severity of the side effects outweighed the clinical benefit to patients with solid tumours [13]. However, this clinical trial did not investigate NQO1 levels in the enrolled patients, nor has any trial testing 17-AAG done so, to the best of our knowledge. This may have important implications as we describe below. Secondly, there are indications in the literature that cultured cell lines may express higher levels of NQO1 than lung and colon patient tumour tissue [14]. We have not been able to directly compare NQO1 levels in cell lines and OSCC tumour tissue, but in vivo expression may well not be as high as that observed in the cultured cell lines.

The findings of this report suggest that if patients could be stratified on the basis of NQO1 protein levels, then OSCC patients expressing NQO1 could potentially benefit from administration of low doses of 17-AAG, possibly in combination with other chemotherapeutics. This is because NQO1 positive patients would likely be responsive to much lower concentrations of the drug. The low dose of 17-AAG would limit the extent of toxic side effects experienced, as observed in clinical trials, where at the six lowest doses administered (6- 125 mg/m^2^), only one out of 20 patients experienced dose-limiting toxicity, compared with eight out of fifteen patients on the two highest doses (175- 225 mg/m^2^) [13]. Since severe hepatotoxicity resulting from 17-AAG treatment is reported to be due to metabolism by a different family of reductases [4], this is unlikely to correlate with NQO1 expression, although this would need to be confirmed in vivo. Furthermore, very low concentrations of 17-AAG would likely have minimal effect on normal cells, even those expressing NQO1, due to their much lower reliance on HSP90. However, there is a clear need for further in vivo testing to confirm that the presence or absence of NQO1 does not affect hepatotoxicity, and that severe side-effects can be mitigated by administration of sufficiently low doses.

An alternative possibility may be the approach proposed by Karkoulis and co-workers [15] for the treatment of bladder cancer. These authors propose that the negative side effects of BA chemotherapeutics (in this case geldanamycin) may be mitigated by orthotopic administration of drug. In the case of OSCC, similar to bladder cancer, the tumour site is relatively accessible; therefore an orthotopic delivery may also be feasible. This would allow exposure to dosages that effectively target the tumour, without reaching systemic concentrations that cause hepatotoxicity.

We noticed that 17-AAG treatment resulted in a dose-dependent decrease in endogenously and exogenously expressed NQO1. This is similar to the effect reported by Gaspar and co-workers [16] who suggested that this down-regulation of NQO1 by 17-AAG may play a role in acquisition of resistance to the drug. We found that there was no down-regulation of NQO1 mRNA levels (Additional file 2: Figure S2), suggesting a post-transcriptional mechanism of control. It is not clear what this mechanism may entail, since disruption of HSP90 activity affects a wide range of cellular functions. Although there is no evidence supporting a direct interaction between NQO1 and HSP90, NQO1 levels are reported to depend heavily on FAD levels [17], which may be disrupted by HSP90 inhibition.

It was interesting to note that the absence of detectable NQO1 in two of the cell lines (WHCO1 and WHCO6) could not be accounted for by the presence of the C609T SNP, but rather seemed to correlate with low expression of the NQO1 gene. Further investigation in tumour samples could shed light on whether this accurately reflects NQO1 expression in patients, or whether it is an artefact of a subset of cultured cell lines. The possibility therefore exists that expression of NQO1 could be induced in these two cell lines under particular environmental circumstances, such as those which may be experienced in cells of a solid tumour, e.g. the presence of reactive oxygen species or hypoxia. We postulate that due to the possibility of induction of the gene in a tumour setting, it will be necessary to specifically investigate NQO1 protein levels in biopsies, in order to estimate potential sensitivity to 17-AAG. This could be done using protein detection (Western blot/immunohistochemistry), or using an NQO1 enzyme activity assay. However, the SNP could be used as a rapid test to exclude patients with a TT genotype, who would not express NQO1 and would therefore be poor candidates for 17-AAG treatment.

The relevance of NQO1 levels in the clinical setting has been discussed by Siegel et al. [18]. The authors make the point that NQO1 levels and activity may not remain stable over the course of the treatment, limiting the predictive value of a protein assay, and supporting use of the SNP as a better biomarker of 17-AAG responsiveness. If the SNP were used as a biomarker for responsiveness, patients with the homozygous null mutation, who will certainly not express active NQO1 could easily be excluded from 17-AAG treatment. While SNP analysis could provide a relatively simple tool for elimination of non-expressors, some patients with the wild-type genotype may also express low levels of the protein, and also be less sensitive to 17-AAG treatment. Thus we propose that 17AAG may still hold promise as a chemotherapy, under certain conditions. These include that the drug either be administered orthotopically, or at low concentrations, using the C609T SNP as a screen to exclude non-expressors of NQO1 who would be poor responders.

## Conclusions

Despite the known side effects of 17-AAG, the extreme sensitivity of NQO1-expressing cell lines to 17-AAG, compared to normal cells or NQO1 negative cells, suggests that this drug could be a useful chemotherapeutic for NQO1 positive OSCC tumours, due to the much lower concentration required for anti-cancer activity. The presence of the C609T SNP in both alleles could be used as a screen to exclude potentially poor responders to 17-AAG treatment at low dosages. This warrants further investigation in an in vivo model.

## Abbreviations

17-AAG: 17-N-allylamino-17-demethoxygeldanamycin; BA: Benzoquinone ansamycin; EGFR: Epidermal growth factor receptor; HSP90: Heat shock protein 90; FAD: Flavin adenine dinucleotide; IGF-1R: Insulin-like growth factor 1 receptor; MTT: 3-(4, 5-dimethylthiazol-2-yl)-2, 5-diphenyltetrazolium bromide; NQO1: NAP (P) H: quinone oxidoreductase 1; OSCC: Oesophageal squamous cell carcinoma; PARP: Poly ADP ribose polymerase; RT-PCR: Real time polymerase chain reaction; RFLP: Restriction fragment length polymorphism; SNP: Single nucleotide polymorphism.

## Competing interests

The authors declare that they have no competing interests.

## Authors’ contributions

KEH performed all the experiments and drafted the manuscript. DTH participated in experimental design and helped draft the manuscript. Both authors read and approved the final manuscript.

## Pre-publication history

The pre-publication history for this paper can be accessed here:

http://www.biomedcentral.com/1471-2407/14/334/prepub

## Supplementary Material

Additional file 1: Figure S1 Expression of NQO1 in stably transfected cells is similar to that of endogenous NQO1 in cell lines in which this is detectable. Whole cell lysates were analysed by western blotting for NQO1, with β-tubulin as a loading control. Click here for file

Additional file 2: Figure S2 17-AAG causes a decrease in endogenous NQO1 levels. Kyse 70 and 150 were treated for 24 h with increasing concentrations of 17-AAG. (A) Cellular protein was harvested and NQO1 levels were determined by Western blotting. β tubulin serves as a loading control. (B) Real time PCR was used to measure levels of NQO1 mRNA relative to the housekeeping gene β actin. Histogram shows pooled results of 2- 4 independent experiments, analysed by one-way ANOVA, but found to be non-significant. Click here for file

Additional file 3: Figure S3 17-AAG causes cell death by apoptosis. (A) Kyse 70 and Kyse 150 cells were treated with 100 nM 17-AAG for different time periods. Total protein lysates were analysed by Western blotting for the presence of cleaved PARP, a marker of apoptosis. β tubulin was used as a loading control. (B) WHCO1 and Kyse 30 were treated with 100 nM 17-AAG for 96 h and whole cell lysates were analysed as described above. Click here for file
