# Supplementary figures and images for: Use of NQO1 status as a selective biomarker for oesophageal squamous cell carcinomas with greater sensitivity to 17-AAG
Source: BMC Cancer. 2014 May 15;14:334. doi: 10.1186/1471-2407-14-334 (PMC4032580; doi:10.1186/1471-2407-14-334)

## Slide 1
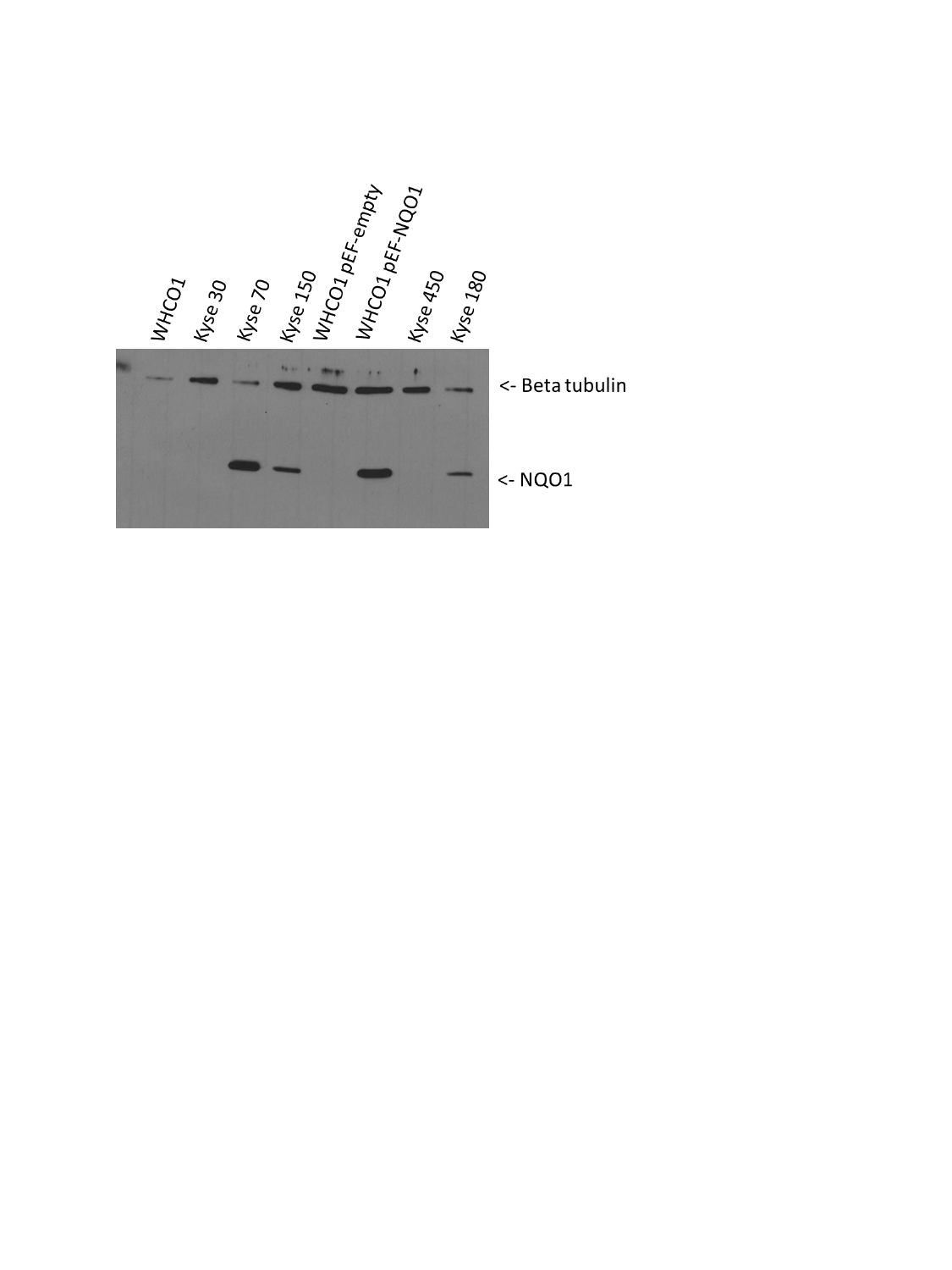

Supplement: Additional file 1: Figure S1 — Expression of NQO1 in stably transfected cells is similar to that of endogenous NQO1 in cell lines in which this is detectable. Whole cell lysates were analysed by western blotting for NQO1, with β-tubulin as a loading control. [file 1471-2407-14-334-S1.pptx]

## Slide 1
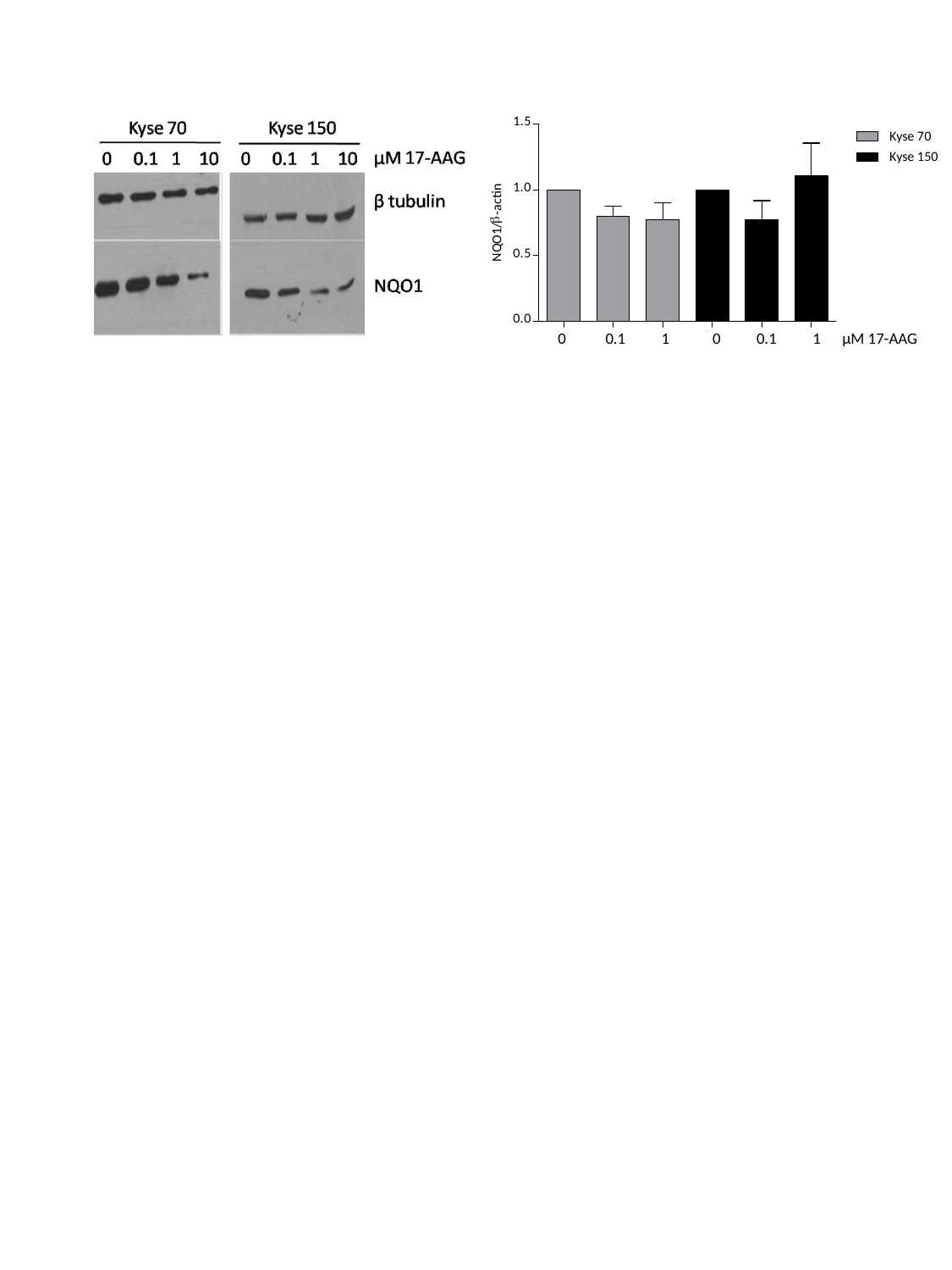

0 0.1 1 0 0.1 1 µM 17-AAG

Supplement: Additional file 2: Figure S2 — 17-AAG causes a decrease in endogenous NQO1 levels. Kyse 70 and 150 were treated for 24 h with increasing concentrations of 17-AAG. (A) Cellular protein was harvested and NQO1 levels were determined by Western blotting. β tubulin serves as a loading control. (B) Real time PCR was used to measure levels of NQO1 mRNA relative to the housekeeping gene β actin. Histogram shows pooled results of 2- 4 independent experiments, analysed by one-way ANOVA, but found to be non-significant. [file 1471-2407-14-334-S2.pptx]

## Slide 1
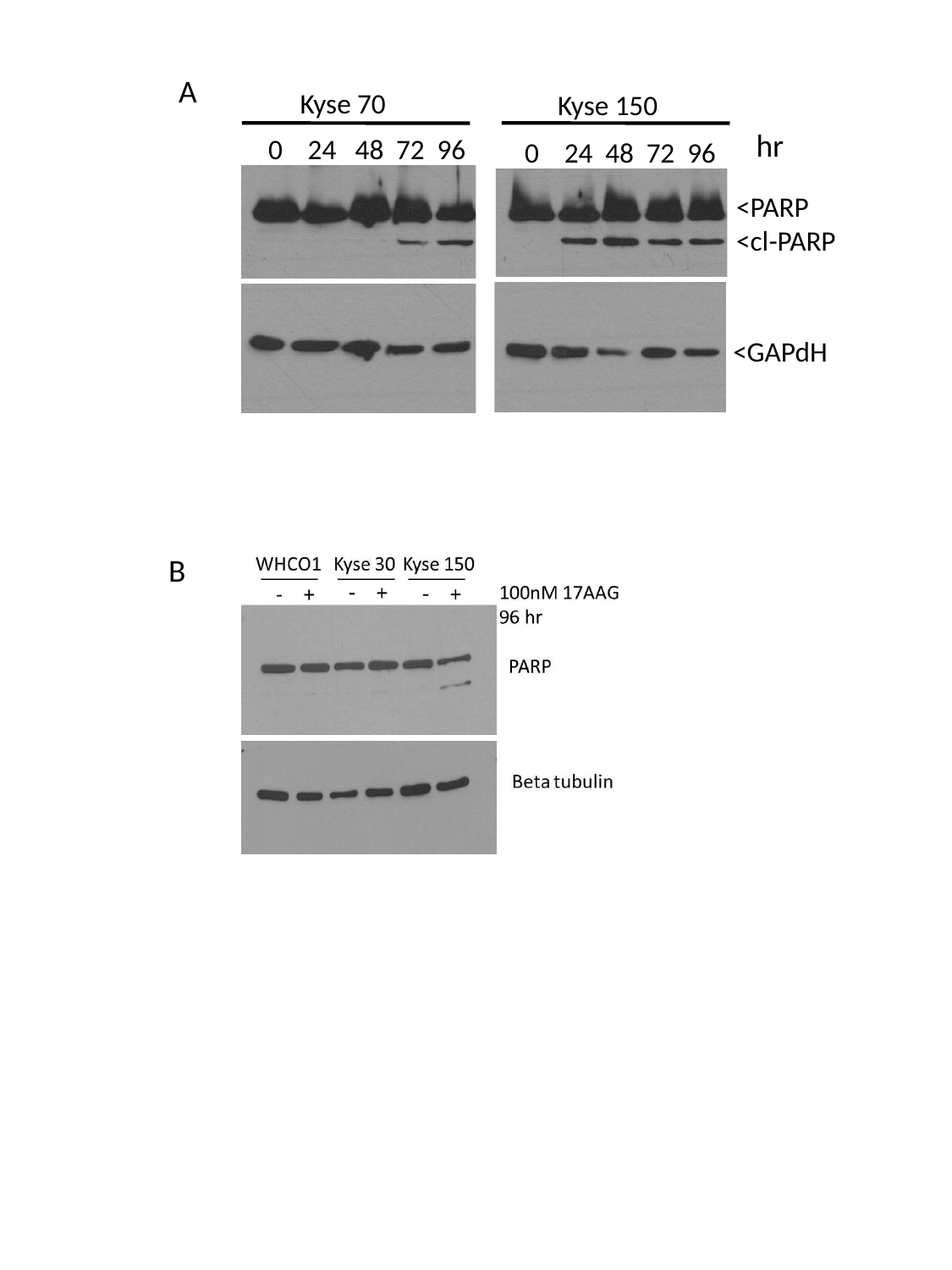

A
Kyse 70
Kyse 150
hr
0 24 48 72 96
0 24 48 72 96
<GAPdH
<PARP
<cl-PARP
B

Supplement: Additional file 3: Figure S3 — 17-AAG causes cell death by apoptosis. (A) Kyse 70 and Kyse 150 cells were treated with 100 nM 17-AAG for different time periods. Total protein lysates were analysed by Western blotting for the presence of cleaved PARP, a marker of apoptosis. β tubulin was used as a loading control. (B) WHCO1 and Kyse 30 were treated with 100 nM 17-AAG for 96 h and whole cell lysates were analysed as described above. [file 1471-2407-14-334-S3.pptx]
